# tidysbml: R/Bioconductor package for SBML extraction into dataframes

**DOI:** 10.1093/bioadv/vbae148

**Published:** 2024-10-03

**Authors:** Veronica Paparozzi, Christine Nardini

**Affiliations:** Institute for Applied Mathematics (IAC) “Mauro Picone”, National Research Council (CNR), Rome 00185, Italy; Institute for Applied Mathematics (IAC) “Mauro Picone”, National Research Council (CNR), Rome 00185, Italy

## Abstract

**Summary:**

We present *tidysbml*, an R package able to perform *compartments*, *species*, and *reactions* data extraction from Systems Biology Markup Language (SBML) documents (up to Level 3) in tabular data structures (i.e. R dataframes) to easily access and handle the richness of the biological information. Thanks to its output format, the package facilitates data manipulation, enabling manageable construction, and therefore analysis, of custom networks, as well as data retrieval, by means of R packages such as *igraph*, *RCy3*, and *biomaRt*. Exemplar data (i.e. SBML files) are extracted from Reactome.

**Availability and implementation:**

The *tidysbml* R package is distributed under CC BY 4.0 License and can be found publicly available in Bioconductor (https://bioconductor.org/packages/tidysbml) and on GitHub (https://github.com/veronicapaparozzi/tidysbml).

## 1 Introduction

The Systems Biology Markup Language (SBML) is a representation format used to describe and store biological processes information in a commonly accepted standard form (Hucka *et al.* 2019). SBML has been launched over 20 years ago and has evolved across three major editions (Levels) that exploit *xml* formatting to provide a detailed and shareable description that facilitates cooperation in the systems biology community and the exchange of models among software applications. Several tools have been developed to support this file format, for instance by providing an interface for different programming languages (e.g. MATLAB/Octave, Python) that enable conversion in graph object, visualization, and analysis tasks. To the best of our knowledge, R incorporates such interfacing with two packages: *SBMLR* and *rsbml*. However, *SBMLR* (Radivoyevitch 2004) provides arrangement of SBML’s data into a list of lists (i.e. an S3 object) up to SBML Level 2, and *rsbml* (Lawrence 2024) exploits the external library *libSBML* to arrange data in an S4 object. As S3 objects with a two-dimensional structure, dataframes are more flexible both for interconnection with other software and within the new grammar being developed in R (the tidyverse). Our package follows this trend, aiming to provide the data structure to receive SBML information that can be further manipulated using the *tidyverse* verbs, and eventually converted in tibble objects when useful. The *tidysbml* package provides conversion of a SBML document (up to Level 3) into a list of R dataframes, each containing information about one of the three main SBML *components* (intuitively named *listOfCompartments*, *listOfSpecies*, *listOfReactions*, see details below), with their biological entities and characteristics. By assigning one row per entity and one column per characteristic, this format enables easy access to SBML data as well as simple manipulation of stored elements. In this way, data can be handled and used as input for other R packages that allow e.g. networks creation, visualization and analysis (e.g. using *igraph* or similar), with possible specialization on regulatory networks [e.g. the *GeneNetworkBuilder* (Ou *et al.* 2024) package that exploits R dataframes in input], or to access and manipulate annotation (e.g. via *biomaRt*-like packages).

In the following, we introduce the *tidysbml* R package, providing its implementation details, a brief description of its main functions, and some examples of application to prove its functionalities for bioinformatics tasks. Beforehand, we give a brief recap on the SBML format to set the terminology and outline how data are being arranged in dataframes.

The SBML description format allows representation of models by organizing information in “components,” each consisting of a list of objects, from here on named “entities”. The main components are the *listOfCompartments*, *listOfSpecies*, and *listOfReactions*. These represent the core of the SBML object, and have remained stable across updates, since SBML Level 1, providing the fundamental information of each biochemical pathway. Each of them provides a similar data structure with three types of elements: (i) *Attributes*, a set of fields determining the properties for each entity (e.g. id, name, metaid); (ii) *Notes*, an element containing human-readable optional information (i.e. a container for descriptions in *XHTML* format); (iii) *Annotation*, a very rich element containing optional data used, for instance, to refer each entity to standard database identifiers. Indeed, SBML uses the Resource Description Framework (RDF) scheme to enable annotations via Universal Resource Identifier (URI) references that are linked to existing publicly available databases and that follow the MIRIAM (Minimal Information Requested in the Annotation of Models) guidelines (Novère *et al.* 2005), providing a standardized format for annotation. Notably, *Annotation* hosts different qualifiers in the form of *xml* tags (e.g. “bqbiol:is,” “bqbiol:hasPart,” “bqbiol:isHomologTo,” “bqbiol:isDescribedBy”). It is hence possible to associate one (if *species* consists of a single molecule) or more identifiers (if the *species* is in fact a molecular complex) after insertion in the “bqbiol:is” and “bqbiol:hasPart” tags, respectively. Similarly, one or more identifiers for homologous entities can be added within the “bqbiol:isHomologTo” tag, and it is also possible to report identifiers for referenced resource, like PubMed (pubmed.ncbi.nlm.nih.gov), using the “bqbiol:isDescribedBy” tag. Moreover, *Annotation* hosts metadata about the SBML document, like creator and author data, time and date of document creation (tags “dcterms:creator” and “dcterms:created,” respectively) or modification (“dcterms:modified” tag). Additional data (kinetic information) are available in SBML to enable not only the analysis of (large) networks, but also the mathematical modelling of (generally small) pathways. Numerous computational frameworks are available to deal with the latter, while this package focuses on the former, within the R language, and for this reason only the three above-mentioned SBML components are processed.

### Implementation

The *tidysbml* R package requires minimal prerequisites for installation. It is entirely written in R and exploits only functions from *base* and *xml2* (Wickham *et al.* 2015) R packages, making it a quick and light package to be installed. It is build and tested to work with R versions ≥4.4, on Linux, Windows and MacOS. More information, including package’s architecture in terms of functions’ dependencies, can be found in last two sections of the Supplementary Material. The package has been accepted in Bioconductor project and can be found publicly available at https://bioconductor.org/packages/tidysbml and on GitHub at https://github.com/veronicapaparozzi/tidysbml.

## 2. Results

The package allows extraction in two main ways: (i) using function as_dfs, which streamlines the process and, after an integrated intermediate conversion of the SBML file into a list (function sbml_as_list), directly outputs four dataframes, or (ii) explicitly calling function sbml_as_list and then as_df to convert the components one by one. The latter can be useful to avoid multiple SBML-to-list conversions, whose complexity is proportional to the SBML file size. The sbml_as_list function uses read_xml and as_list functions from *xml2* to read and transform the SBML file into a list of lists suitably nested according to xml’s tags format. This aims at improving navigation and extraction of xml’s data by browsing an R list rather than an R xml object. Specially, the function enables conversion for both the entire SBML model in input (setting the second argument as “all”) and the model restricted to one “listOf” level (e.g. *listOfSpecies*), to focus on a specific type of entity only. See Vignette and R documentation for further information.

### 2.1 Dataframes structure

For all the three *components* (i.e. *listOfCompartments*, *listOfSpecies*, and *listOfReactions*), the resulting dataframes are composed by a number of columns corresponding to the number of items defined in each element (*Attributes*, *Notes*, and *Annotation*), i.e. if the tag is never defined, the column is not created. Column naming is standardized using: (i) *Attribute’*s names directly, (ii) “notes” for *Notes*; (iii) *Annotation’*s qualifier prefixed by “annotation_” (e.g. the “annotation_hasPart” column reports the “bqbiol:hasPart” content). Whenever one tag consists of multiple elements (e.g. multiple identifiers for *Annotation*, or multiple paragraphs for *Notes*), elements are delimited by “ ” (i.e. single-space character) for *Annotation* tag, or by “|” (i.e. pipe character) for *Notes*. Given an SBML file, the full extraction with our package returns conversion of the three components into a list of four dataframes (i.e. *df_compartments*, *df_species*, *df_reactions*, *df_species_in_reactions*), namely one for *compartments*, one for *species*, and two for *reactions* data, as *listOfReactions* provides information for both reactions and the species there involved, resulting in two additional column for *df_species_in_reactions*, reporting the “reaction_id” and the species’ role inside the reaction (i.e. Reactant, Product, or Modifier).

### 2.2 Applications

In the following, we present *tidysbml* functionalities by showing use cases on the Reactome “Aryl hydrocarbon receptor signalling” pathway (R-HSA-8937144), chosen as a real world example of limited size easy to visualize, and the Reactome “Cell-Cell communication” pathway (R-HSA-1500931), chosen to have a conspicuous example for the *species* id’s conversion. Due to the broad panorama of possible applications, we will focus only on some of the many examples allowed, in order to show the possibilities in general without specializing to particular uses or representations (as can happen for networks). For this reason, the code for the various transformations is explicitly reported without implementing specific functions. We refer the reader to the Supplementary Material for accessing the code reproducing the examples in R.

**Figure 1.**
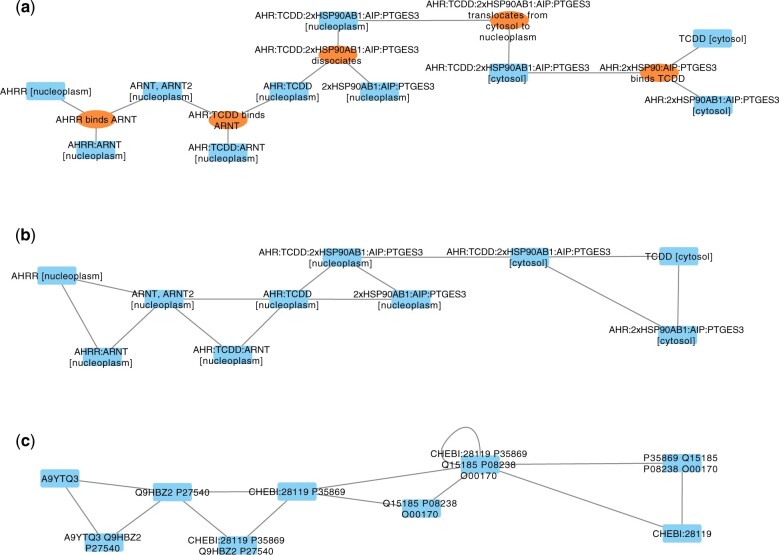
Examples of graph visualization in Cytoscape via *RCy3* package versus standard Cytoscape import from Reactome’s SBML (R-HSA-8937144). Blue rectangles are nodes for species, orange ovals are nodes representing reactions. (a) Cytoscape output with SBML input: standard Cytoscape network when importing from SBML file, reactions are represented as nodes. (b) *RCy3* R function output with *igraph* input: Cytoscape network imported from a custom graph build in R from an *edgelist* whose nodes are labeled after *tidysbml* labels extraction from SBML (see edgelist_personalized1 in Supplementary Material). (c) *RCy3* R function output with a dataframe input: Cytoscape network imported from a custom graph build in R using an *edgelist* whose nodes labels are MIRIAM ids, as provided by *tidysbml* (see edgelist_personalized2 in Supplementary Material), in this case there are only UniProt and ChEBI ids.

The first example concerns network visualization and analysis, by means of *igraph* (Csárdi *et al.* 2024) and *RCy3* (Gustavsen *et al.* 2019) R packages, illustrated in Fig. 1. The objective is to use SBML’s data in R to build networks, specifically custom networks. With *tidysbml*, it is possible to create a simple *igraph* graph (Fig. 1a) starting from *species* and *reactions* data (see Section 1.1 in Supplementary Material for R code), that are provided by the dataframe with information about which species are involved in each reaction, namely *df_species_in_reactions*. Further, it is possible to create a customized graph exploiting the amount of information contained in SBML documents. One example is to create columns for personalized *edgelist* (two-column table) using information reported in dataframes, for instance considering *species* as nodes and linking though arcs them when they belong to the same *reaction*. Figure 1b shows such a type of graph with nodes labeled by *species*’ SBML names, while Fig. 1c shows the graph obtained from the same SBML file, but whose *species*’ names contains the “annotation” tag concatenated MIRIAM ids (see also Section 1.2 of Supplementary Material); this latter manipulation is extremely effective under this dataframe structure. Note the power and flexibility of *tidysbml*: Fig. 1c’s structure is slightly different from Fig. 1b’s one, where AHR:TCDD: 2xHSP90AB1:AIP:PTGES3 appears twice since the *compartment* information is preserved (one node is in the nucleoplasm and one in the cytosol), while in Fig. 1c, compartments have been discarded, hence the two nodes are merged and their connection is properly recognized as a loop. *tidysbml* can just as easily preserve the *compartment* name and recreate a network with the same structure of Fig. 1c (see Section 1.2 in Supplementary Material for this alternative result).

On the other hand, there exist also bioinformatic software tools, which provide network analysis and visualization, like Cytoscape (Shannon *et al.* 2003). Packages such as *RCy3* permit communication between R and the Cytoscape environment using different R network classes (e.g. “igraph,” “graphNEL” networks) or dataframes. In this way, it is possible to exploit Cytoscape analysis tools after re-creating Cytoscape networks starting from an *igraph* network or directly using an *edgelist* in form of dataframe, as manipulated *tidysbml* dataframes offer (see Section 2 in Supplementary Material). For the sake of completeness, we notice that it is also possible to load SBML files either directly in Cytoscape or in R through *RCy3*; however, the resulting network presents one node for each *species*/*reaction* and one edge linking each *species* to the *reaction* in which it is involved, thus missing the potential for digraphs representation and all the richness of information (*Notes*, *Annotation* data) typical of the SBML standard, that are conversely preserved in *tidysbml*.

The second example concerns species names conversions, a popular task in bioinformatics that can be achieved via several (online) tools. Nevertheless, this approach requires different steps of copy/paste, import and export, which is the motivation for the creation of packages like *biomaRt* (Durinck *et al.* 2005). The usage of *tidysbml* coupled with *biomaRt* streamlines the conversion task with simple matching of the appropriate column retrieving URIs reported in SBML documents. In the following example, we extract URIs from “annotation_is” column (containing SBML data in the “bqbiol:is” tag) in *df_species*. In particular, after conversion through *tidysbml*, URIs data can be extracted from each row of the *listOfSpecies* dataframe, split and collected inside a single structure (e.g. R vector) and eventually filtered to retain only selected types of URIs (e.g. UniProt). Then, the ID portion from each URI can be held with regular expression manipulation and saved in a final vector used to filter the *biomaRt* dataframe (see Section 3 and Fig. S1 of Supplementary Material for detailed R code).

## 3 Conclusions

The *tidysbml* is a light package that allows extraction of SBML information about *compartments*, *species* and *reactions* (i.e. the content of *listOfCompartments*, *listOfSpecies*, *listOfReactions* tags) in a dataframe format. The purpose of this package is to enable the management of SBML data that can be used for different (large) network analyses. In fact, the output data structure is particularly effective for accessing and querying data, allowing easy manipulation through general *dplyr* verbs or specific converters like the *biomaRt* package and further transformation in network structures (by means of R packages like *igraph* and *RCy3*), overall facilitating these and more fundamental tasks in systems biology, as these (non exhaustive) use cases have the ambition to show.

## Supplementary Material

vbae148_Supplementary_Data

## Data Availability

The data underlying this article are available in Reactome Pathway Database at https://reactome.org. SBML documents can be accessed using 8937144 and 1500931 identifiers (“Aryl hydrocarbon receptor signalling” and “Cell-Cell communication” pathways, respectively).
