# Supplementary material for: tidysbml: R/Bioconductor package for SBML extraction into dataframes
Source: Bioinform Adv. 2024 Oct 3;4(1):vbae148. doi: 10.1093/bioadv/vbae148 (PMC11479578; doi:10.1093/bioadv/vbae148)
Supplement: vbae148_Supplementary_Data [file vbae148_supplementary_data.pdf]

# tidysbml: R/Bioconductor package for SBML extraction into dataframes
Supplementary Material

Veronica Paparozzi, Christine Nardini

This Supplementary Material provides the R code to perform the tasks described in the "Results" section of the *tidysbml* main document. In the following, each section reports the code for integrating *tidysbml* with other R packages, namely *igraph* [1], *RCy3* [2] and *biomaRt* [3]. Finally, we report in the last two sections information about package's performances and functions' interdependencies.

## 1 Implementation examples with *igraph*

This section describes two cases of the usage of *igraph* graphs created via manipulation of *tidysbml*'s dataframes, both using the same SBML file (Reactome R-HSA-8937144) [4]. The first one is a relatively straightforward network built using ready-to-use information in the *tidysbml* dataframes; the second one, instead, handles dataframe's information in order to show two further examples of custom network construction. These (visualized after importing in Cytoscape via *RCy3*) are depicted in the *tidysbml* principal document at Fig. 1b and Fig. 1c.

### 1.1 Simple network

The following code creates *igraph* graph using information about the *species* involved in each *reaction*, namely data from the second dataframe in the third component of the `as_dfs()` output list, i.e. *df_species_in_reactions*. The code describes the *tidysbml* steps for dataframes extraction, then the *dplyr* [5] commands for simple data manipulation, and finally the *igraph* function for network creation:

```
# 1. tidysbml
library(tidysbml)
filepath <- system.file("extdata", "R-HSA-8937144.sbml", package = "tidysbml")
list_dfs <- as_dfs(filepath, type = "file")

# 2. dplyr
library(dplyr)
df_species_reactions <- list_dfs[[3]][[2]]
    %>% select("reaction_id", "species") #source-target columns

# 3. igraph
library(igraph)
graph <- graph_from_data_frame(df_species_reactions)
plot(graph)
```

### 1.2 Personalized networks

It is possible to use dataframes' information for creation of different networks. Here we build a network having *species* as nodes and arcs whenever two species are involved in the same *reaction*. In particular, two examples of personalized *edgelist* (i.e. dataframe with two columns for "source" and "target" nodes; each pair defines one (directed) arc) are described: the first one defines a network where nodes are labeled according to the "name" column in the dataframe of species (i.e. *df_species*), while the second one has labels according to *Annotation* columns containing for each species (i.e. node) its

(concatenated) MIRIAM ids. To reproduce the examples, the following codes for the *edgelists* creation replace the *dplyr* chunk in the previous subsection.
Nodes labeled using their name (Fig. 1b), as reported within the SBML document inside the *name* attribute exploiting *tidysbml* extraction:

```
library(dplyr)
df <- list_dfs[[3]][[2]]
        %>% left_join(list_dfs[[2]], by=c("species"="id"))
        %>% select("reaction_id", "name")
nr <- nrow(df)
col_source <- rep(NA,nr) #initialize edgelist first column
col_target <- rep(NA,nr) #initialize edgelist second column
k <- 0 #initialize edgelist index
# create edgelist's columns
for(i in seq_len(nr-1)){
    for (j in c((i+1):nr)){
        if(df$reaction_id[i]==df$reaction_id[j]){
            k <- k+1
            col_source[k] <- df$name[i]
            col_target[k] <- df$name[j]
        }
    }
}
# create edgelist
edgelist_personalized1 <- data.frame("source" = na.omit(col_source),
                                     "target" = na.omit(col_target))
```

The following lines can be appended to the former to obtain the results in the second example (Fig. 1c), with nodes renamed using concatenated MIRIAM ids contained in "annotation_is" and "annotation_hasPart" columns:

```
df <- list_dfs[[2]]
# 1. create vector with clean ids
vec_nameids <- vapply(seq_along(df$annotation_hasPart), function(i){
    if(is.na(df$annotation_hasPart[i])){
        vec_tmp <- unlist( strsplit(df$annotation_is[i], " ", fixed = TRUE) )
        idx <- match(-1, unlist(gregexpr("reactome", vec_tmp)))
        vec_tmp <- vec_tmp[idx]
    } else {
        vec_tmp <- unlist( strsplit(df$annotation_hasPart[i], " ", fixed = TRUE))
    }
    paste( vapply(vec_tmp, function(x){
                chr1 <- "/"
                first <- max(unlist(gregexpr(chr1, x)))
                substr(x, first + 1, nchar(x))
            }, FUN.VALUE = character(1)),
            collapse = " ")
}, FUN.VALUE = character(1))
# 2. create dataframe for join
dim_species <- data.frame("id" = df$id, "name" = df$name, "miriam_ids"= vec_nameids)
# 3. create edgelist
edgelist_personalized2 <- left_join(edgelist_personalized1,dim_species, by=c("source"="name"))
                            %>% left_join(dim_species, by=c("target"="name"))
                            %>% select(miriam_ids.x, miriam_ids.y)
                            %>% `colnames<-`(c("source", "target"))
```

Since `edgelist_personalized2` does not retain information about species' location (i.e. *compartment*) within the species' label, providing in this way a different type of network (see Fig. 1c) respect to the

one obtained with `edgelist_personalized1` (Fig. 1b), we show that it is also possible to include this information and, thus, obtaining a network with the same number of nodes and edges, by replacing the following lines instead of the second snippet in the previous chunk, namely the line for `dim_species` definition:

```
dim_species <- data.frame("id" = df$id, "name" = df$name, "miriam_ids"= vec_nameids,
                          "compartment_id"=df$compartment)
dim_compartments <- data.frame("id"=list_dfs[[1]]$id, "name"=list_dfs[[1]]$name)
dim_species <- left_join(dim_species, dim_compartments, by=c("compartment_id"="id"))
                %>% select(name.x, miriam_ids, name.y)
                %>% `colnames<-`(c("name", "miriam_ids", "compartment_name"))
                %>% mutate("miriam_ids" = paste0(miriam_ids," [",compartment_name,"]"))
```

Then, after creating the *edgelist*, the corresponding *igraph* graph can be build and visualized, similarly to the third chunk in the example in Section 1.1:

```
graph_p <- igraph::graph_from_data_frame(edgelist_personalized2, directed = FALSE)
plot(graph_p)
```

# 2 Implementation with *RCy3*

This implementation can be used starting from either an *igraph* graph or an *edgelist* table. The first type requires only one *RCy3* function call (i.e. `createNetworkFromIgraph`), while the second one needs also creation of a suitable dataframe. Here R code for the second type of example is shown, to be appended to the second chunk of code in Section 1.1 (i.e. after the definition of `df_species`$_r$*eactions*) :

```
colnames(df_species_reactions) <- c("source","target")
RCy3::createNetworkFromDataFrames(edges = df_species_reactions)
```

# 3 Implementation with *biomaRt*

This section provides code for accessing *biomaRt* information starting from the SBML data contained in the *tidysbml*'s output dataframes.

After conversion via *tidysbml*, similarly to the first example but starting from another SBML file (R-HSA-1500931) [6],

```
filepath <- system.file("extdata", "R-HSA-1500931.sbml", package = "tidysbml")
list_of_dfs <- as_dfs(filepath, type = "file")
```

we can select the dataframe of *species*

```
df_species <- list_of_dfs[[2]]
```

and split the URIs using as delimiter " " into a vector `vec` of URIs

```
vec <- na.omit( unlist( lapply(df_species$annotation_is, function(x){
    unlist( strsplit(x, " ", fixed = TRUE))
})))
```

We can also filter and retain only one type of URI, for instance UniProt [7] URIs, as follows

```
vec_unip <- na.omit( unlist( lapply(X = vec, FUN = function(x){
        if( all(unlist(gregexpr("uniprot", x)) > -1)){
            x
        } else {
            NA
        }
    })
))
```

| | uniprot_gn_id | entrezgene_id | external_gene_name | entrezgene_description |
|---|---|---|---|---|
| 1 | O00241 | 10326 | SIRPB1 | signal regulatory protein beta 1 |
| 2 | O00401 | 8976 | WASL | WASP like actin nucleation promoting factor |
| 3 | O14936 | 8573 | CASK | calcium/calmodulin dependent serine protein kinase |
| 4 | O15117 | 2533 | FYB1 | FYN binding protein 1 |
| 5 | O43914 | 7305 | TYROBP | transmembrane immune signaling adaptor TYROBP |
| 6 | O60500 | 4868 | NPHS1 | NPHS1 adhesion molecule, nephrin |
| 7 | O60716 | 1500 | CTNND1 | catenin delta 1 |
| 8 | O75563 | 8935 | SKAP2 | src kinase associated phosphoprotein 2 |
| 9 | P03950 | 283 | ANG | angiogenin |
| 10 | P05556 | 3688 | ITGB1 | integrin subunit beta 1 |

Figure S1: Example conversion from UniProt to NCBI ids through *biomaRt* package, showing a portion of `df_mart_uniprot` dataframe as visualized in RStudio. First column ("uniprot_gene_id") contains the UniProt ids available on *biomaRt*, second column ("entrezgene_id") the NCBI ids, third ("external_gene_name") and fourth ("entrezgene_description") columns are for further information about each entity, that are name and description respectively.

and finally create a vector with the corresponding UniProt ids using these simple manipulations

```
vec_ids <- vapply( vec_unip, function(x){
                    chr1 <- "/"
                    first <- max(unlist(gregexpr(chr1, x)))
                    substr(x, first + 1, nchar(x))
                  }, FUN.VALUE = character(1))
```

After these steps of data cleaning, we can execute *biomaRt* commands

```
library(biomaRt)
mart <- useEnsembl(biomart = "ensembl", dataset = "hsapiens_gene_ensembl")
```

and use the output vector `vec_ids` to, e.g., look at the conversion of UniProt into NCBI [8] ids (i.e. "entrezgene_id" column):

```
df_mart_uniprot <- getBM(attributes = c("uniprot_gn_id", "entrezgene_id",
                            "external_gene_name", "entrezgene_description"),
                         filters = "uniprot_gn_id",
                         values = vec_ids,
                         mart = mart)
```

An example of output dataframe is shown in Fig. S1.

# 4 Package performances

The *tidysbml* package has been tested on different machines using different SBML documents. Here we report results for full extraction of the biggest document only, thus showing solely the worst performance detected. The principal machines used for testing are (i) Windows PC (CPU: AMD FX-6300, 6 cores; RAM: 8gb) and (ii) MacBook Air (Chip: Apple M1; RAM: 8gb). The largest document used for testing (i.e. R-HSA-168256 [9], size 15.5MB) required about 20 seconds for full extraction performed by the former machine and about 6 seconds on the latter (with the greatest part of the running time required for execution of `as_list` function from *xml2*). The majority of the other tested documents are smaller in size (few megabytes) requiring consequentially shorter running times. During development and testing, no particular minimal requirements for machine performances have been highlighted.

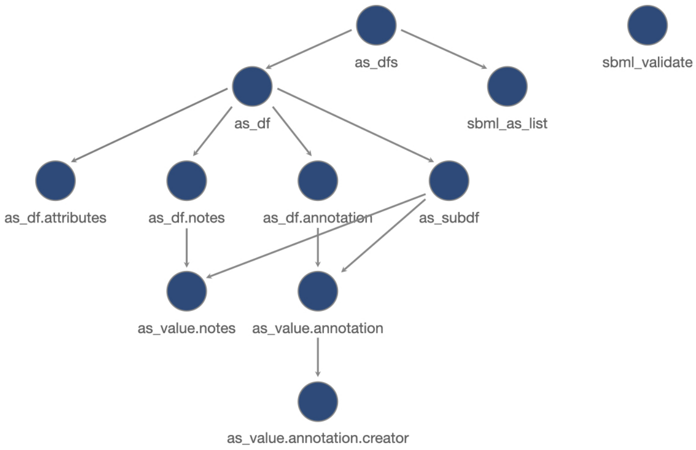


Figure S2: Network representation of the interdependencies among functions' package. Nodes are the package's functions, arrows point to the functions that are called by the starting one.

# 5 Representation of functions' interdependencies

In this section we report the network diagram representing the dependency relationships among functions within the *tidysbml* package, namely how package's functions depends one another in terms of 'calls'. In Fig. S2 is shown the network where nodes represent functions and edges are directed by pointing to the function that is called, namely the function in the tail node calls the function in the head node (e.g. `as_dfs` calls both `as_df` and `sbml_as_list`). No dependencies result in no links (e.g. see the `sbml_validate` node).

# References

[1] Csárdi, G., Nepusz, T., Traag, V., Horvát, S., Zanini, F., Noom, D., Müller, K. igraph: Network Analysis and Visualization in R. (2024), https://doi.org/10.5281/zenodo.7682609, R package version 2.0.3, https://CRAN.R-project.org/package=igraph

[2] Gustavsen, A. J, Pai, Shraddha, Isserlin, Ruth, Demchak, Barry, Pico, R. A. RCy3: Network Biology using Cytoscape from within R. (2019), *F1000Research*, https://doi.org/10.12688/f1000research.20887.3

[3] Durinck, S., Moreau, Y., Kasprzyk, A., Davis, S., De Moor, B., Brazma, A., Huber, W. BioMart and Bioconductor: a powerful link between biological databases and microarray data analysis. (2005), *Bioinformatics (Oxford, England)*, 21(16), 3439–3440. https://doi.org/10.1093/bioinformatics/bti525

[4] [dataset]* Jassal, B. "Aryl hydrocarbon receptor signalling". Reactome, release 87, URL with StableID: R-HSA-8937144 (2024-06-19)

[5] Wickham, H., François, R., Henry, L., Müller, K., Vaughan, D. dplyr: A Grammar of Data Manipulation. (2023), R package version 1.1.4, https://dplyr.tidyverse.org, https://github.com/tidyverse/dplyr

[6] [dataset]* Garapati, P. V. "Cell-Cell communication". Reactome, release 89, URL with StableID: R-HSA-1500931 (2024-06-19)

[7] The UniProt Consortium. UniProt: the Universal Protein Knowledgebase in 2023. (2023) Nucleic Acids Research, 51(D1), D523–D531, https://doi.org/10.1093/nar/gkac1052

[8] Sayers, E. W., Bolton, E. E., Brister, J. R., Canese, K., Chan, J., Comeau, D. C., Connor, R., Funk, K., Kelly, C., Kim, S., Madej, T., Marchler-Bauer, A., Lanczycki, C., Lathrop, S., Lu, Z., Thibaud-Nissen, F., Murphy, T., Phan, L., Skripchenko, Y., Tse, T., Wang, J., Williams, R., Trawick, B.W., Pruitt, K.D., Sherry, S. T. Database resources of the national center for biotechnology information. (2022) Nucleic acids research, 50(D1), D20–D26, https://doi.org/10.1093/nar/gkab1112

[9] [dataset]* Ouwehand, W.H., Luo, F., de Bono, B., Gillespie, M.E. ”Immune System”. Reactome, release 89, URL with StableID: R-HSA-168256 (2024-06-19)
